# Right main pulmonary artery distensibility on dynamic ventilation CT and its association with respiratory function

**DOI:** 10.1186/s41747-024-00441-5

**Published:** 2024-04-04

**Authors:** Tatsuya Oki, Yukihiro Nagatani, Shota Ishida, Masayuki Hashimoto, Yasuhiko Oshio, Jun Hanaoka, Ryo Uemura, Yoshiyuki Watanabe

**Affiliations:** 1 Department of Radiology, Shiga University of Medical Science, Seta-Tsukinowa-Cho, Otsu, Shiga 520-2192 Japan; 2 Department of Radiological Technology, Kyoto College of Medical Science, 1-3 Sonobecho Oyamahigashimachi Imakita, Nantan, Kyoto, 622-0041 Japan; 3 Department of Thoracic Surgery, Takeda General Hospital, 28-1 Ishida Moriminamicho, Fushimi-Ku, Kyoto, 601-1434 Japan; 4 Division of General Thoracic Surgery, Department of Surgery, Shiga University of Medical Science, Seta-Tsukinowa-Cho, Otsu, Shiga 520-2192 Japan

**Keywords:** Hypertension (pulmonary), Pulmonary artery, Pulmonary disease (chronic obstructive), Tomography (x-ray computed), Ventilation (Fourier analysis)

## Abstract

**Background:**

Heartbeat-based cross-sectional area (CSA) changes in the right main pulmonary artery (MPA), which reflects its distensibility associated with pulmonary hypertension, can be measured using dynamic ventilation computed tomography (DVCT) in patients with and without chronic obstructive pulmonary disease (COPD) during respiratory dynamics. We investigated the relationship between MPA distensibility (MPAD) and respiratory function and how heartbeat-based CSA is related to spirometry, mean lung density (MLD), and patient characteristics.

**Methods:**

We retrospectively analyzed DVCT performed preoperatively in 37 patients (20 female and 17 males) with lung cancer aged 70.6 ± 7.9 years (mean ± standard deviation), 18 with COPD and 19 without. MPA-CSA was separated into respiratory and heartbeat waves by discrete Fourier transformation. For the cardiac pulse-derived waves, CSA change (CSAC) and CSA change ratio (CSACR) were calculated separately during inhalation and exhalation. Spearman rank correlation was computed.

**Result:**

In the group without COPD as well as all cases, CSACR exhalation was inversely correlated with percent residual lung volume (%RV) and RV/total lung capacity (*r* = -0.68, *p* = 0.003 and *r* = -0.58, *p* = 0.014). In contrast, in the group with COPD, CSAC inhalation was correlated with MLDmax and MLD change rate (MLDmax/MLDmin) (*r* = 0.54, *p* = 0.020 and *r* = 0.64, *p* = 0.004) as well as CSAC exhalation and CSACR exhalation.

**Conclusion:**

In patients with insufficient exhalation, right MPAD during exhalation was decreased. Also, in COPD patients with insufficient exhalation, right MPAD was reduced during inhalation as well as exhalation, which implied that exhalation impairment is a contributing factor to pulmonary hypertension complicated with COPD.

**Relevance statement:**

Assessment of MPAD in different respiratory phases on DVCT has the potential to be utilized as a non-invasive assessment for pulmonary hypertension due to lung disease and/or hypoxia and elucidation of its pathogenesis.

**Key points:**

• There are no previous studies analyzing all respiratory phases of right main pulmonary artery distensibility (MPAD).

• Patients with exhalation impairment decreased their right MPAD.

• Analysis of MPAD on dynamic ventilation computed tomography contributes to understanding the pathogenesis of pulmonary hypertension due to lung disease and/or hypoxia in patients with expiratory impairment.

**Graphical Abstract:**

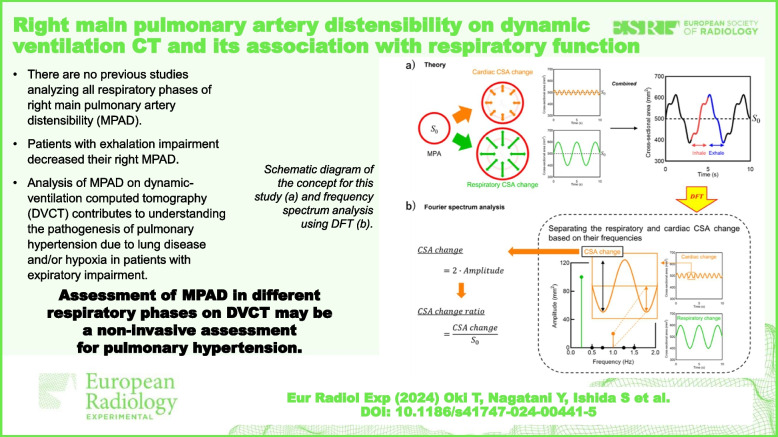

## Background

Chronic obstructive pulmonary disease (COPD) is an often progressive inflammatory disease of the airways, the alveoli, and the microvasculature. The airway abnormalities of chronic bronchitis and the peripheral loss of parenchymal lung texture in emphysema are probably caused not only by already known risk factors such as inhaled particles and gases from cigarette smoking and biomass fuel but also by diverse cellular and pathophysiological changes with distinct genetic backgrounds [1]. COPD is a leading cause of morbidity and mortality worldwide, and various intra- and extrapulmonary complications exist, but pulmonary hypertension due to lung disease and/or hypoxia, which is classified as group 3 in the Nice classification, is associated with higher mortality [2]. Several previous studies have investigated pulmonary artery distensibility in pulmonary hypertension [3–6]. However, these may not reflect physiological hemodynamics because they were measured with breath-hold at end-inspiration or even if they were evaluated under respiration, they did not distinguish between inspiration and expiration.

Dynamic chest radiography enables observation of respiratory dynamics, and the mean lung density changes over time were considered to be a composite wave of respiration and heartbeat, so these were separated into respiratory and heartbeat waves for analyzing cardiac kinetics in respiratory dynamics [7, 8]. However, the heartbeat waves in these studies were superimposed in the anterior-posterior direction, and they did not evaluate the blood vessels themselves directly.

On the other hand, the recent clinical application of iterative reconstruction has made it possible to dramatically reduce radiation dose on chest computed tomography (CT) while maintaining the detectability of lung nodules [9–13]. Four-dimensional dynamic ventilation CT (DVCT), which is performed continuously while the patient breathes at rest, has expanded from research to clinical applications. So far, findings have been obtained regarding pleural adhesion assessment, airway and peripheral imbalance, lung field torsion, and pleural motion [14–21]. However, only one paper examining the drop heart has been published on the cardiovascular system [22], and no papers assessing pulmonary vasculature have been published to date.

In this study, to evaluate right main pulmonary artery distensibility (MPAD) in patients with and without COPD during respiratory dynamics, we measured cross-sectional area (CSA) change on DVCT and separated the obtained waves into respiratory-derived and heart pulsation-derived waves using discrete Fourier transformation (DFT). Then, we clarified the relationship between MPAD and airflow limitation and between MPAD and expiratory impairment by examining how the heart pulsation-derived wave, which reflects MPAD, was related to mean lung density, spirometry, and patient background.

## Methods

This study was approved by the institutional review board of our institution, with written informed consent obtained from all patients. This research was retrospectively performed as an additional evaluation after our previous research, which focused on evaluating the detectability of DVCT for localized pleural adhesion with independent quantification of both the pleura and chest wall during respiration [15, 16].

### Subjects

**Fig. 1:**
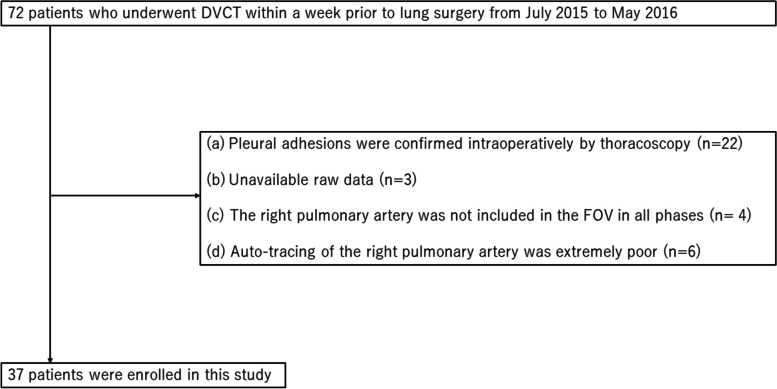
Patient enrollment. DVCT, Dynamic ventilation computed tomography; FOV, Field of view

Among 72 patients who underwent DVCT within 1 week prior to lung surgery between July 2015 and May 2016, considering the impact of heterogeneity of lung field mechanical stress within respiratory motion caused by the presence and distribution of pleural adhesions on pulmonary artery distensibility, we excluded 22 cases in which pleural adhesions were confirmed by intraoperative thoracoscopy. Raw data was unavailable in 3 cases. In addition, we excluded cases in which the right MPA (MPA) was not included in the field of view in all phases, and 43 cases were extracted. Of these, 6 cases with significantly poor auto-tracing (described below) were excluded, leaving a total of 37 cases analyzed retrospectively (Fig. 1). Finally, the total study population was classified into 18 patients with COPD and 17 those without based on a standardized spirometric pulmonary function test in accordance with the American Thoracic Society guideline [23]. The 18 COPD patients were classified into 11 patients with COPD to a mild degree (Global Initiative for Chronic Obstructive Lung Disease [GOLD] stage 1, forced expiratory volume in 1 s [FEV_1_]/forced vital capacity [FVC] < 0.7, and FEV_1_ predicted > 0.8), 6 patients with COPD to a moderate degree (GOLD stage 2, FEV_1_/FVC < 0.7, and 0.5 < FEV_1_ predicted < 0.8), and 1 patient with COPD to a severe degree (GOLD stage 3, FEV_1_/FVC < 0.7 and 0.3 < FEV_1_ predicted < 0.5). Table 1 summarizes the patients’ characteristics.

**Table 1:** Patient characteristics and significant differences between the groups in patients with and without chronic obstructive pulmonary disease (COPD)

Variable	With COPD	Without COPD	*p*-value
Number	18	19	–
Age	73.3 ± 5.9	68.0 ± 8.9	0.039*
Females/males	10/8	10/9	0.893
Body mass index	22.1 ± 3.3	24.4 ± 3.2	0.020*
Smoking index	784.3 ± 623.8	581.1 ± 602.5	0.284

^*^p < 0.05

Some study subjects have been previously included in one of our previous studies [15, 16, 20, 21, 24] in the following way. In the selection process for the study population in the present study, 32 patients were identified among 72 patients who were enrolled between July 2015 and May 2016 in our previous study for different purposes: assessment of the detectability of localized benign pleural adhesion [15, 16, 24], continuous measurement of the main bronchial dimensions and lung density in the lateral position for smokers [20], and evaluation of the association of respiratory functional indices and smoking with pleural movement [21].

### Four-dimensional CT protocol

Each patient underwent preoperative routine protocol CT, followed approximately 10 min later by DVCT with the following protocol; in most of the patients, 28 of 37, contrast-enhanced CT was performed, and the remaining 9 patients underwent non-contrast CT. First, a scanning range of 16 cm in the *z*-axis direction was set as the field of view to include all target lesions in the lungs, and the patient was asked to breathe according to a predefined breathing cycle [15, 16, 20, 21]. Dynamic image data were acquired using DVCT for 7.04 ± 1.33 s including at least 1 breath on a 320-row CT (Canon Medical Systems, Otawara, Tochigi, Japan) in wide-volume scan mode.

Data acquisition parameters on DVCT were as follows: tube current, 20 mA; tube voltage, 120 kVp; rotation time, 0.35 s; field of view, 320 mm; collimation, 0.5 mm; and slice thickness, 0.5 mm. Reconstruction parameters were as follows: collimation, 0.5 mm; slice thickness, 0.5 mm; standard reconstruction kernel, FC13; 1 frame interval, 0.35 s; and full reconstruction method. The effective dose was calculated by multiplying the dose-length product value based on the CT dose index quantity by a factor of 0.017 [25].

### Post-processing

DVCT images were transferred to a dedicated workstation (PhyZiodynamics; Ziosoft, Tokyo, Japan) for post-processing. This software uses a motion coherence function that interpolates motion between phases to create interphase motion by generating three more phases between the original phases and produces a quadruple-complemented image to obtain smooth four-dimensional motion [26]. This function was used to improve the accuracy of the Fourier transformation described below to create data with apparently high temporal resolution.

### Image analysis: measurement

**Fig. 2:**
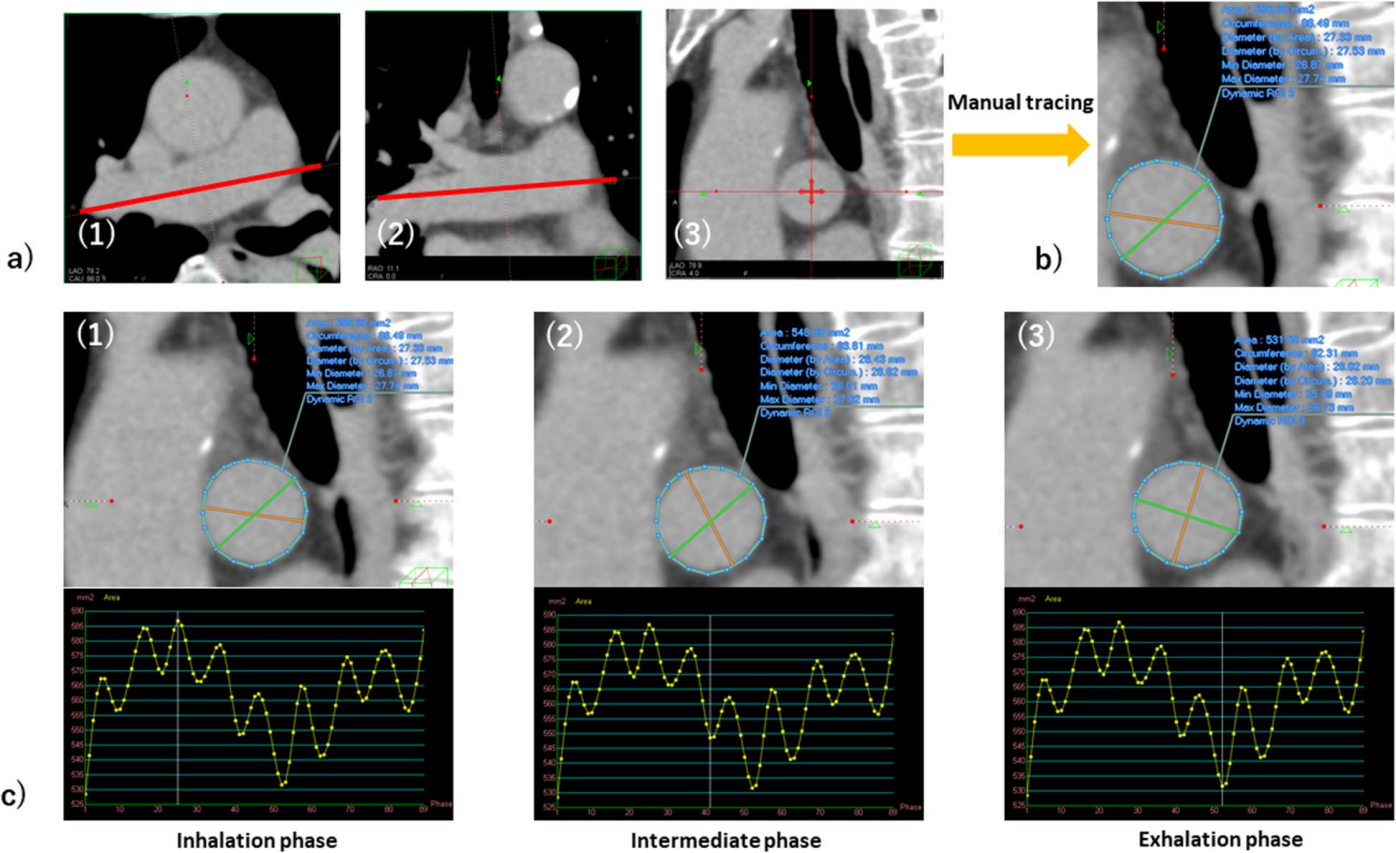
The phase of maximal inhalation was visually identified from respiratory motion, and oblique axial/coronal images parallel to the right MPA were created at that phase (**a** (1, 2) red line). In the oblique sagittal image, we determined the cross-section located exactly in the middle of the right MPA; from the trunk of the pulmonary artery—the right MPA bifurcation to the right MPA—right superior pulmonary artery bifurcation (**a** (3)). We manually traced the right MPA as finely as possible in this cross-section (**b**). This was followed by auto-tracing to the remaining phases, *e.g.,* inhalation phase (**c** (1)), intermediate phase (**c** (2)), and exhalation phase (**c** (3)). The cross-sectional area (mm^2^) of the right MPA in each phase was then output. MPA, Main pulmonary artery

A total of three board-certified diagnostic radiologists with 5, 8, and 23 years of experience were involved in this process. We visually identified the phase of maximal inhalation based on respiratory motion and created an oblique sagittal image that was orthogonal to the right MPA in that phase. In the oblique sagittal image, we determined the cross-section located exactly in the middle of the right MPA: from the right MPA bifurcation to the right superior pulmonary artery bifurcation (Fig. 2a). In this cross-section, the right MPA was manually traced as finely as possible (Fig. 2b), and this was followed by automatic tracing in the remaining phases (Fig. 2c). In addition, we measured the CT value of the MPA lumen in the identical section. We checked to see if the automated tracing was adequate and excluded six cases in which it was deemed inadequate. The CSA of the right MPA (mm^2^) in each phase was then output. The above process was measured by two board-certified diagnostic radiologists with five and 23 years of experience.

The mean lung density (MLD) was automatically measured at each time frame using dedicated software (Lung Volume Measurement; Canon Medical Systems), and MLD changes over time were plotted. The expiratory peak MLD (MLDmax), inspiratory peak MLD (MLDmin), and MLD change rate (MLD CR = MLDmax/MLDmin) were determined from the plot. The above process was measured by two board-certified diagnostic radiologists with 8 and 23 years of experience.

In addition, we evaluated the patient’s emphysematous changes visually on conventional CT acquired at peak inspiration, taken before the DVCT using the Goddard Classification Score for depicting emphysematous areas as low attenuation areas. Briefly, emphysema severity was scored with a 4-grade scale: 0 points (emphysematous change extending to < 5% of the lung field) to 4 points (≥ 75%) in each of six areas: upper (aortic arch level), middle (carina level), and lower lung field (upper-end diaphragm level), and summed; the maximal possible score is 24 [27]. This process was measured by two board-certified diagnostic radiologists with 5 and 23 years of experience in consensus.

### Data conversion

**Fig. 3:**
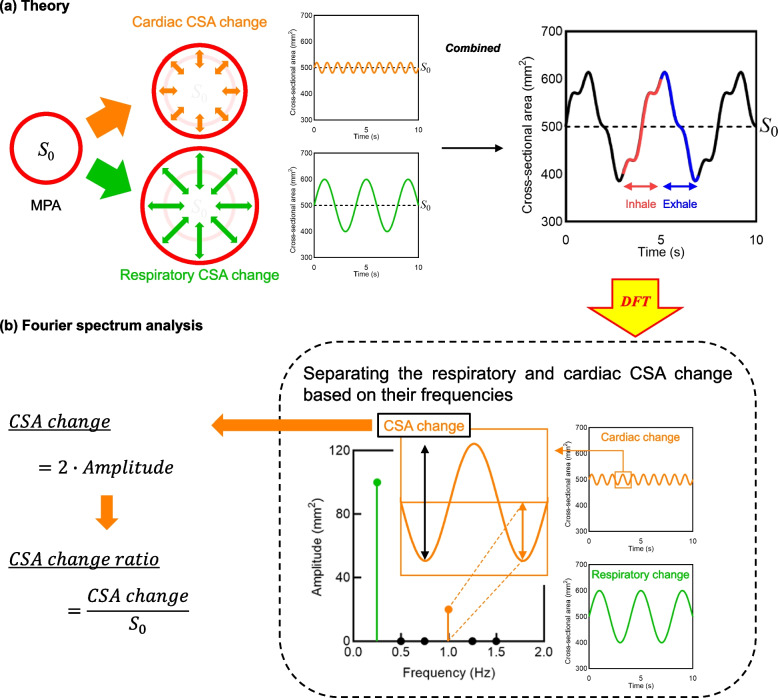
Schematic diagram of the concept for this study (**a**) and frequency spectrum analysis using DFT (**b**). Temporal changes in CSA of the right MPA are derived from respiration and cardiac pulsation. DVCT measures the combined wave of respiratory- and cardiac pulsation-derived changes in MPA-CSA (**a**). DFT was used to extract the cardiac pulsation-derived MPA-CSA change from the measured waveforms, because DFT can separate the combined wave into individual frequency waves (**b**). First, DFT was performed on MPA-CSA waveforms measured using DVCT. Subsequently, the amplitude of the waveform was quantified from the DFT complex data by calculating their absolute values. Then, the cardiac pulsation-derived components were extracted based on the heart rate. The cardiac pulsation-derived MPA-CSA changes were defined as peak-to-bottom of the waveforms, *i.e.,* twice the waveform amplitude. Finally, the change ratio of MPA-CSA was calculated by dividing the cardiac-pulsation-derived MPA-CSA changes by the direct-current component of the DFT components (*S*_0_) to eliminate patient-specific anatomical MPA-CSA differences. The direct current component indicates the MPA-CSA without respiratory- and cardiac pulsation-derived waves, which corresponds to the mean value of MPA-CSA along all scan phases. These procedures were performed for the data determined during the inhalation and exhalation phases, respectively. CSA, Cross-sectional area; DFT, Discrete Fourier transformation; DVCT, Dynamic ventilation computed tomography; MPA, Main pulmonary artery

As Fig. 3a shows, temporal changes in MPA-CSA were combinations of respiratory- and cardiac pulsation-derived changes with different frequencies [8]. Thus, the measured data using DVCT was the combined wave of respiratory- and cardiac pulsation-derived changes. Frequency spectrum analysis using DFT, which can separate combined waves into individual frequency waves, has been widely applied for analyzing biological functions, particularly using magnetic resonance images [28, 29]. Therefore, this study employed DFT to separate MPA-CSA changes into respiratory- and cardiac pulsation-derived changes based on their frequencies (Fig. 3).

First, DFT was performed for MPA-CSA waves measured during the inhalation and exhalation phases. Subsequently, frequency components corresponding to heart rate were extracted as the cardiac pulsation-derived component. Heart rate was visually determined from the MPA-CSA waveforms by consensus of the two board-certified diagnostic radiologists with 5 and 23 years of experience. It should be noted that all heart rates were normal. Then, the change ratio of MPA-CSA was calculated by dividing the cardiac-pulsation-derived MPA-CSA changes by the direct-current component of the DFT components (*S*_0_ in Fig. 3) to eliminate patient-specific anatomical MPA-CSA differences. It is worth noting that the direct-current component indicates the MPA-CSA without respiratory- and cardiac pulsation-derived waves, which corresponded to the mean value of MPA-CSA along all scan phases. Finally, the following six values were calculated: CSA changes during the inhalation phase (CSAC In), CSA change ratio during the inhalation phase (CSACR In), CSA changes during the exhalation phase (CSAC Ex), CSA change ratio during the exhalation phase (CSACR Ex), the maximal value of CSA during both inhalation and exhalation phase (CSAmax), and the minimal value of CSA during both inhalation and exhalation phase (CSAmin). We used these values as indicators to evaluate the right MPAD.

### Pulmonary function evaluation

Within 1 month from the date of the DVCT examination, preoperative spirometry (residual lung volume [RV], %RV, total lung capacity [TLC], RV/TLC, forced expiratory volume in 1 s [FEV_1_]/forced vital capacity [FVC], FEV_1_ predicted, etc.) was performed on all eligible patients in accordance with the American Thoracic Society criteria, with RV, %RV, TLC, and RV/TLC as measures of expiratory impairment, and FEV_1_/FVC, FEV_1_ predicted were used as measures of airflow limitation.

### Statistical analysis

Significant differences between patients with and without COPD were analyzed using the Mann–Whitney* U* test for MPA-CSA (*S*_0_ In, *S*_0_ Ex, CSAmax, CSAmin, CSAC In, CSACR In, CSAC Ex, and CSACR Ex), Goddard Classification Score, spirometry (RV, %RV, TLC, RV/TLC, FEV_1_/FVC, and FEV_1_ predicted), mean lung density on CT (MLDmax, MLDmin, and MLD CR), and patient information (age, height, and body weight). The same items were also analyzed using Spearman rank correlation as well as coefficients of determination among all patients, patients with COPD, and patients without COPD. In addition, a cross-correlation between the two board-certified diagnostic radiologists with 5 and 23 years of experience was determined for the CSA measurements. The statistical significance level was set at 0.05. We used IBM SPSS Statistics 25.0 (IMB, Chicago, IL, USA) as the software for these statistical analyses.

## Results

A significant difference was found in age between patients with and those without COPD, as shown in Table 1 (*p* = 0.039). However, no significant difference was found between age and measured values on DVCT such as CSA and MLD, between age and Goddard Classification Score, and between age and spirometric results (*p* > 0.05). In addition, BMI in patients with COPD was lower than that in those without (*p* = 0.020). Although BMI did not correlate with CSA, BMI correlated positively with total lung capacity (*r* = 0.375, *p* = 0.026), MLDmax (*r* = 0.471, *p* = 0.023), and MLDmin (*r* = 0.361, *p* = 0.028).

Table 2 demonstrates patients’ data of spirometry, conventional CT, and DVCT for all patients and patients with and without COPD. It compares these data between patients with and without COPD. FEV_1/_FVC, FEV_1_ predicted, MLDmax, and MLD CR were all higher in patients without COPD.

**Table 2:** Mean values of measurements in spirometry, DVCT, and conventional CT in all patients and the patients with and without COPD and differences between the patients with and without COPD

		Total	With COPD	Without COPD	*p*-value
DVCT	*S*_0_ In	491.8 ± 115.7	485.4 ± 131.8	497.8 ± 97.5	0.715
	S Ex	496.0 ± 118.9	489.7 ± 133.5	502.0 ± 101.7	0.605
	CSAmax	510.8 ± 120.6	504.4 ± 136.1	516.9 ± 103.4	0.523
	CSAmin	477.6 ± 114.7	471.3 ± 130.2	483.7 ± 97.4	0.715
	CSAC In	19.2 ± 12.8	19.4 ± 13.6	19.0 ± 12.6	0.940
	CSAC Ex	23.2 ± 19.6	26.3 ± 25.5	20.4 ± 11.7	0.753
	CSACR In	8.0 ± 5.3	8.5 ± 6.4	7.6 ± 4.2	0.940
	CSACR Ex	9.6 ± 7.4	11.2 ± 9.6	8.1 ± 4.1	0.599
	MLDmax	-740.7 ± 40.6	-756.9 ± 28.5	-725.3 ± 44.8	0.012*
	MLDmin	-816.1 ± 29.6	-821.9 ± 28.7	-810.6 ± 30.1	0.210
	MLD CR	0.093 ± 0.035	0.079 ± 0.029	0.106 ± 0.036	0.020*
Conventional CT	Goddard	2.68 ± 5.04	4.44 ± 6.08	1.00 ± 3.13	0.031*
Spirometry	RV	2.03 ± 0.53	2.05 ± 0.46	2.01 ± 0.61	0.454
	%RV	109.8 ± 21.3	109.3 ± 19.5	110.2 ± 23.5	0.973
	TLC	5.14 ± 1.12	5.19 ± 1.05	5.08 ± 1.22	0.708
	RV/TLC	0.397 ± 0.060	0.400 ± 0.068	0.395 ± 0.054	0.708
	FEV_1_/FVC	69.8 ± 11.1	61.3 ± 8.2	77.8 ± 6.6	0.000*
	FEV_1_ predicted	91.3 ± 15.6	83.9 ± 16.3	98.3 ± 11.2	0.005*

*COPD* Chronic obstructive pulmonary disease, *CSAC Ex* Change of cross-sectional area during the exhalation phase, *CSAC In* Change of cross-sectional area during the inhalation phase, *CSACR Ex* Change ratio of cross-sectional area during the exhalation phase, *CSACR In* Change ratio of cross-sectional area during the inhalation phase, *CSAmax* Maximal value of CSA during both inhalation and exhalation phases, *CSAmin* Minimal value of CSA during both inhalation and exhalation phases, *DVCT* Dynamic ventilation computed tomography, *FEV*_*1*_ Forced expiratory volume in 1 s, *FVC* Forced vital capacity, *Goddard* Goddard Classification Score, *MLD CR* Change ratio in the mean lung density, *RV* Residual volume, *S*_*0*_* Ex S*_0_ During the exhalation phase, *S*_*0*_* In S*_0_ During the inhalation phase, *TLC* Total lung capacity

Data are given as mean ± standard deviation. *S*_0_ is defined as the direct-current component of the DFT components

^*^p < 0.05

The relationships between CSA and spirometry, between CSA and MLD, and between CSA and Goddard Classification Score are shown in Table 3. In all cases, CSA during the exhalation phase (CSAC Ex, CSACR Ex) showed inverse correlations with both %RV and RV/TLC, and CSACR Ex also showed an inverse correlation with RV. CSA during the inhalation phase (CSAC In, CSACR In) did not show these relationships. Similar to the relationship in all patients, in patients without COPD, CSAC Ex was inversely correlated with %RV, and CSACR Ex was inversely correlated with RV, %RV, TLC, and RV/TLC, but none of these correlated with MLD. In patients with COPD, on the other hand, CSAC Ex, CSACR Ex, and CSAC In were correlated with MLDmax and MLD CR.

**Table 3:** Spearman’s rank correlations and coefficients of determination for CSA and spirometry or CSA and MLD or CSA and Goddard Classification Score for all cases and in patients with and without COPD

			RV	%RV	TLC	RV/TLC	EFV_1_/FVC	FEV_1_ predicted	MLDmax	MLDmin	MLD CR	Goddard
Total	CSAC In	*r*	-0.032	-0.283	0.044	-0.200	0.073	-0.098	0.183	0.095	0.163	-0.243
		*p*-value	*0.858*	*0.104*	*0.801*	*0.258*	*0.666*	*0.563*	*0.278*	*0.577*	*0.335*	*0.147*
		*R*^2^	0.004	0.046	0.003	0.031	0.006	0.001	0.042	0.015	0.028	0.006
	CSAC Ex	*r*	-0.301	**-0.443**	-0.049	**-0.377**	0.039	0.038	0.266	0.099	0.257	-0.126
		*p*-value	*0.083*	0.009*	*0.778*	0.028*	*0.820*	*0.825*	*0.111*	*0.561*	*0.124*	*0.456*
		*R*^2^	0.048	0.153	0.000	0.118	0.018	0.012	0.060	0.035	0.031	0.101
	CSACR In	*r*	-0.085	-0.228	0.021	-0.246	0.086	-0.138	0.149	0.055	0.144	-0.231
		*p*-value	*0.635*	*0.194*	*0.903*	*0.161*	*0.612*	*0.415*	*0.380*	*0.746*	*0.394*	*0.169*
		*R*^2^	0.000	0.003	0.005	0.016	0.006	0.003	0.019	0.005	0.015	0.010
	CSACR Ex	*r*	**-0.385**	**-0.472**	-0.055	**-0.505**	0.025	0.006	0.234	0.030	0.256	-0.064
		*p*-value	0.024*	0.005*	*0.756*	0.002*	*0.883*	*0.971*	*0.162*	*0.860*	*0.126*	*0.708*
		*R*^2^	0.014	0.055	0.000	0.062	0.015	0.012	0.035	0.022	0.017	0.072
With COPD	CSAC In	*r*	-0.052	-0.407	0.105	-0.275	0.234	0.056	**0.542**	0.001	**0.639**	-0.139
		*p*-value	*0.844*	*0.105*	*0.680*	*0.286*	*0.349*	*0.826*	0.020*	*0.997*	0.004*	*0.584*
		*R*^2^	0.002	0.046	0.014	0.042	0.042	0.023	0.356	0.000	0.469	0.000
	CSAC Ex	*r*	-0.284	-0.446	0.157	-0.404	0.317	0.170	**0.540**	0.154	**0.498**	0.037
		*p*-value	*0.268*	*0.073*	*0.533*	*0.107*	*0.200*	*0.499*	0.021*	*0.542*	0.035*	*0.885*
		*R*^2^	0.034	0.175	0.015	0.159	0.001	0.002	0.399	0.114	0.151	0.162
	CSACR In	*r*	0.055	-0.176	0.143	-0.132	0.290	0.063	**0.490**	0.028	**0.575**	-0.111
		*p*-value	*0.833*	*0.498*	*0.572*	*0.613*	*0.243*	*0.804*	0.039*	*0.913*	0.013*	*0.860*
		*R*^2^	0.017	0.007	0.033	0.007	0.127	0.035	0.260	0.001	0.323	0.008
	CSACR Ex	*r*	-0.178	-0.306	0.244	-0.434	0.368	0.187	0.424	0.086	0.416	0.053
		*p*-value	*0.495*	*0.232*	*0.329*	*0.082*	*0.132*	*0.458*	*0.079*	*0.735*	*0.086*	*0.834*
		*R*^2^	0.004	0.009	0.050	0.054	0.018	0.000	0.314	0.101	0.107	0.081
Without COPD	CSAC In	*r*	-0.102	-0.277	-0.116	-0.211	0.056	-0.318	-0.075	0.119	-0.246	-0.316
		*p*-value	*0.697*	*0.282*	*0.656*	*0.417*	*0.819*	*0.185*	*0.759*	*0.627*	*0.311*	*0.187*
		*R*^2^	0.010	0.054	0.002	0.021	0.003	0.097	0.000	0.055	0.050	0.075
	CSAC Ex	*r*	-0.372	**-0.500**	-0.379	-0.341	0.033	-0.004	0.100	0.000	0.174	-0.424
		*p*-value	*0.142*	0.041*	*0.134*	*0.181*	*0.892*	*0.986*	*0.684*	*1.000*	*0.477*	*0.070*
		*R*^2^	0.183	0.269	0.120	0.081	0.001	0.003	0.011	0.003	0.013	0.033
	CSACR In	*r*	-0.331	-0.336	-0.180	-0.419	-0.065	-0.447	-0.177	0.088	-0.316	-0.298
		*p*-value	*0.194*	*0.188*	*0.489*	*0.094*	*0.792*	*0.055*	*0.468*	*0.721*	*0.188*	*0.215*
		*R*^2^	0.047	0.080	0.016	0.076	0.000	0.181	0.005	0.028	0.072	0.083
	CSACR Ex	*r*	**-0.626**	**-0.681**	**-0.506**	**-0.581**	0.009	-0.078	0.096	-0.075	0.254	-0.284
		*p*-value	0.007*	0.003*	0.038*	0.014*	*0.972*	*0.751*	*0.694*	*0.759*	*0.293*	*0.238*
		*R*^2^	0.352	0.428	0.235	0.188	0.018	0.006	0.011	0.000	0.026	0.012

*COPD* Chronic obstructive pulmonary disease, *CSAC Ex* Change of cross-sectional area during the exhalation phase, *CSAC In* Change of cross-sectional area during the inhalation phase, *CSACR Ex* Change ratio of cross-sectional area during the exhalation phase, *CSACR In* Change ratio of cross-sectional area during the inhalation phase, *FEV*_*1*_ Forced expiratory volume in 1 s, *FVC* Forced vital capacity, *Goddard* Goddard Classification Score, *MLD CR* Change ratio in the mean lung density, *RV* Residual volume, *TLC* Total lung capacity, *r* Spearman rank correlation coefficient, *R*^*2*^ coefficients of determination

For association with statistically significant correlation, *r*-value and *p*-value are demonstrated in bold normal and bold italic styles, respectively

^*^p < 0.05

The relationship between spirometry and MLD and between spirometry and Goddard Classification Score is shown in Table 4. %RV and MLDmax were inversely correlated in all cases and in patients with COPD. Goddard Classification Score correlated negatively with FEV_1_/FVC in all cases and in patients with COPD.

**Table 4:** Spearman rank correlations and coefficients of determination for spirometry and MLD and Goddard Classification Score for all cases and in patients with and without COPD

			RV	%RV	TLC	RV/TLC	EFV_1_/FVC	FEV_1_ predicted
Total	MLDmax	*r*	-0.235	**-0.365**	-0.065	-0.268	0.312	0.132
		*p*-value	*0.182*	0.034*	*0.712*	*0.126*	*0.060*	*0.435*
		*R*^2^	0.032	0.111	0.010	0.040	0.094	0.042
	MLDmin	*r*	-0.023	-0.086	0.005	-0.044	0.094	-0.049
		*p*-value	*0.898*	*0.627*	*0.975*	*0.805*	*0.579*	*0.774*
		*R*^2^	0.000	0.008	0.001	0.000	0.006	0.001
	MLD CR	*r*	-0.309	**-0.422**	-0.133	-0.326	**0.334**	0.271
		*p*-value	*0.075*	0.013*	*0.448*	*0.060*	0.043*	*0.104*
		*R*^2^	0.067	0.141	0.012	0.070	0.132	0.101
	Goddard	*r*	0.069	-0.104	0.270	-0.168	**-0.430**	**-0.401**
		*p*-value	*0.699*	*0.560*	*0.116*	*0.344*	0.008*	0.014*
		*R*^2^	0.005	0.102	0.048	0.151	0.174	0.101
With COPD	MLDmax	*r*	-0.481	**-0.642**	-0.267	-0.466	0.236	0.095
		*p*-value	*0.051*	0.005*	*0.284*	*0.060*	*0.345*	*0.708*
		*R*^2^	0.227	0.220	0.043	0.140	0.147	0.132
	MLDmin	*r*	-0.348	-0.145	-0.384	-0.034	0.168	-0.173
		*p*-value	*0.171*	*0.580*	*0.116*	*0.896*	*0.505*	*0.491*
		*R*^2^	0.132	0.080	0.140	0.006	0.062	0.001
	MLD CR	*r*	-0.189	-0.346	0.075	-0.400	0.040	0.316
		*p*-value	*0.468*	*0.174*	*0.769*	*0.112*	*0.874*	*0.202*
		*R*^2^	0.022	0.054	0.031	0.122	0.026	0.150
	Goddard	*r*	0.000	-0.087	0.400	-0.285	**-0.575**	-0.464
		*p*-value	*1.000*	*0.741*	*0.100*	*0.267*	0.013*	*0.052*
		*R*^2^	0.017	0.165	0.113	0.300	0.246	0.091
Without COPD	MLDmax	*r*	0.055	-0.257	0.119	-0.100	-0.221	-0.176
		*p*-value	*0.833*	*0.319*	*0.649*	*0.701*	*0.363*	*0.470*
		*R*^2^	0.001	0.112	0.000	0.007	0.065	0.071
	MLDmin	*r*	0.353	0.120	0.430	0.123	**-0.472**	-0.225
		*p*-value	*0.164*	*0.646*	*0.085*	*0.639*	0.041*	*0.355*
		*R*^2^	0.108	0.004	0.097	0.008	0.261	0.125
	MLD CR	*r*	-0.274	**-0.556**	-0.243	-0.233	0.018	-0.064
		*p*-value	*0.288*	0.020*	*0.348*	*0.368*	*0.943*	*0.794*
		*R*^2^	0.120	0.300	0.089	0.041	0.008	0.004
	Goddard	*r*	0.057	-0.136	0.175	-0.066	0.360	0.006
		*p*-value	*0.828*	*0.604*	*0.501*	*0.802*	*0.130*	*0.980*
		*R*^2^	0.002	0.084	0.002	0.033	0.080	0.014

*COPD* Chronic obstructive pulmonary disease, *FEV*_*1*_ Forced expiratory volume in 1 s, *FVC* Forced vital capacity, *Goddard* Goddard Classification Score, *MLD* Mean lung density, *RV* Residual volume, *TLC* Total lung capacity,* r* Spearman’s rank correlation coefficient, *R*^*2*^ coefficients of determination

For association with statistically significant correlation, *r*-value and *p*-value are demonstrated in bold normal and bold italic styles, respectively

^*^p < 0.05

The CT density value of the MPA in the phase of maximal inhalation was 89.9 ± 30.3 HU. The dose length product (DLP) value was 8.06 mGy cm for a single rotation, and the total estimated radiation exposure for DVCT was 2.74 ± 0.52 mSv.

## Discussion

This study showed that the right MPAD during exhalation was reduced when exhalation was insufficient, in other words, when residual volume remained larger at the peak expiration associated with expiratory impairment during physiologic respiratory kinetics. This was observed in all patients and a subgroup analysis of patients without COPD, but in patients with COPD, MPAD was reduced not only during exhalation but also during inhalation, suggesting that more severe expiratory impairment may affect MPAD during both inhalation and exhalation.

**Fig. 4:**
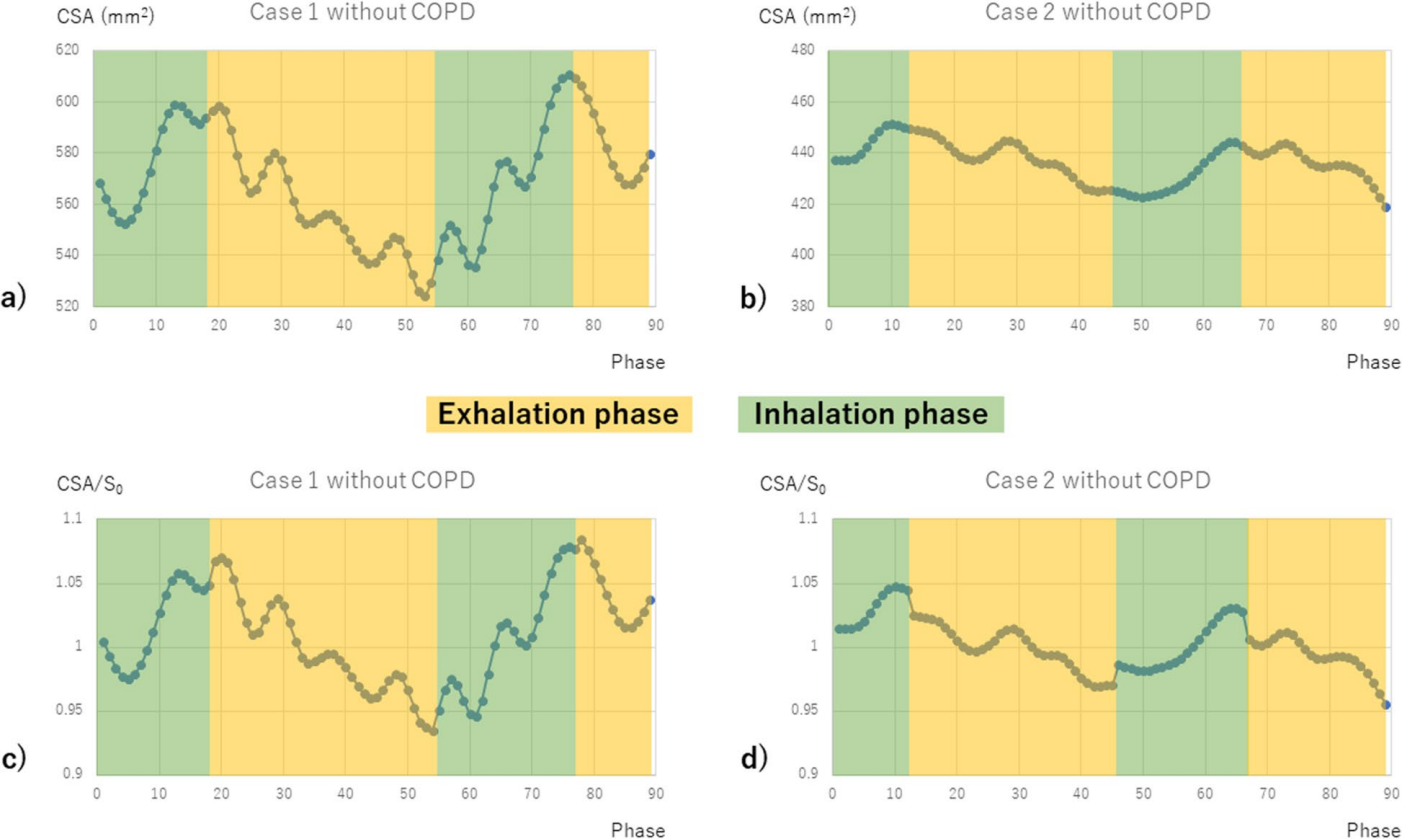
CSA changes in two patients without COPD are shown in the four graphs, where the horizontal axis is the respiratory phase, the vertical axis in images (**a** and **b**) is CSA (mm^2^), and the vertical axis in images (**c** and **d**) is CSA/*S*_0_, which is the direct-current component of the DFT components. Case 1 had low residual air volume (%RV 91.8, RV/TLC 0.366); case 2 had high residual air volume (%RV 116.5, RV/TLC 0.428). In the latter case with high residual air volume, the decrease in CSA during exhalation was more pronounced than during inhalation. COPD, Chronic obstructive pulmonary disease; CSA, Cross-sectional area; RV, Residual lung volume; TLC, Total lung capacity

**Fig. 5:**
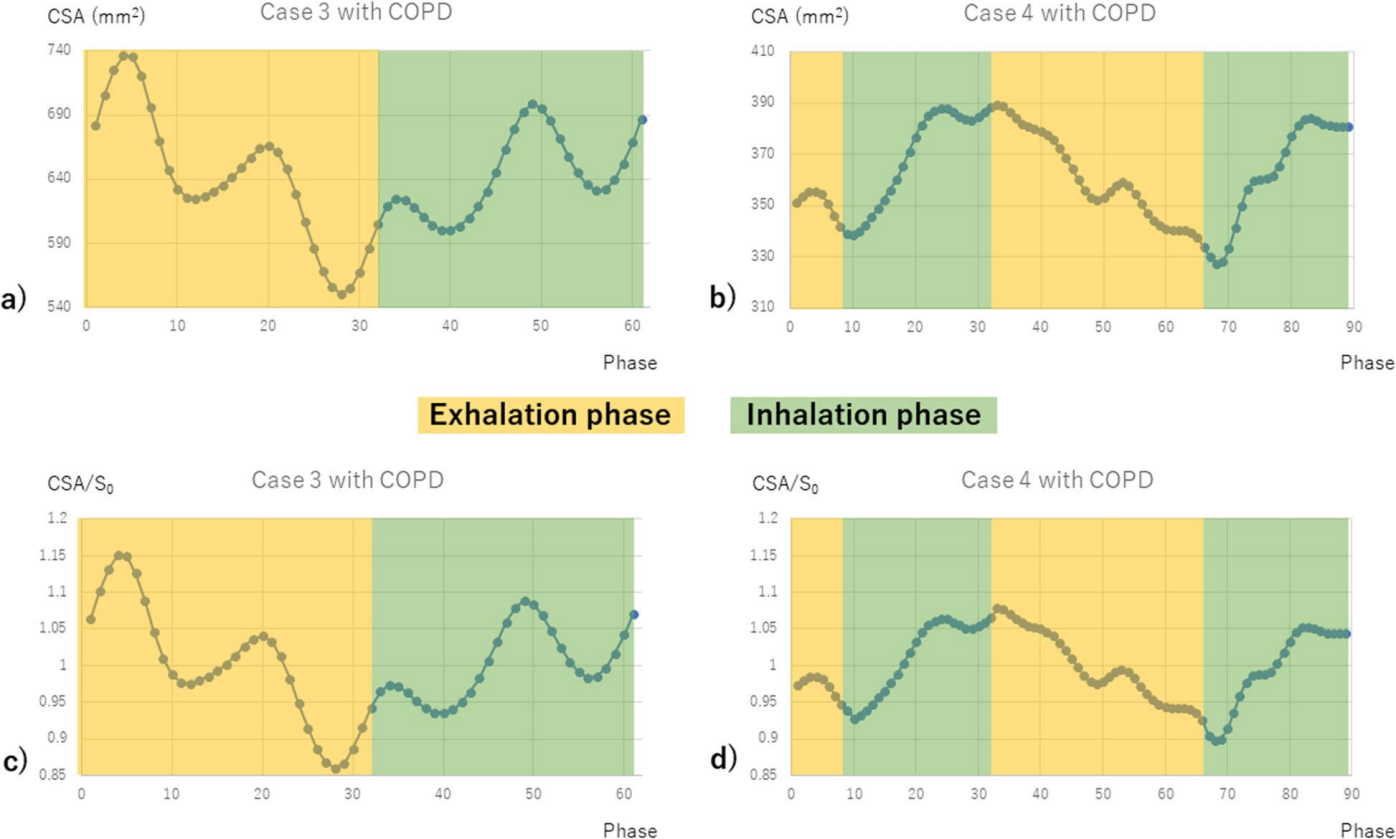
CSA changes in two patients with COPD are shown in the four graphs, where the horizontal axis is the respiratory phase, the vertical axis in images (**a** and **b**) is CSA (mm^2^), and the vertical axis in images (**c** and **d**) is CSA/*S*_0_, which is the direct current component of the DFT components. Case 3 had a high MLD with MLDmax -698.6 and MLD CR 0.106, and case 4 had a low MLD with MLDmax -831.2 and MLD CR 0.041. In the latter case, where the MLD was low, CSA decreased not only on exhalation but also on inhalation. COPD, Chronic obstructive pulmonary disease; CSA, Cross-sectional area; MLD, Mean lung density; RV, Residual lung volume; TLC, Total lung capacity

The CSA changes due to heartbeat during inhalation and exhalation represent the average change in that respiratory phase, which reflects MPAD. Overall, insufficient exhalation, *i.e.,* high residual air volume (higher %RV and RV/TLC), decreased MPAD during exhalation. This result suggested that in cases of insufficient exhalation, intrathoracic pressure during exhalation became relatively higher, resulting in a lower MPAD. When subgroup analysis was performed according to the presence or absence of airflow limitation, in patients without COPD, as in the whole, MPAD decreased during exhalation when residual air volume is high (*e.g.*, Fig. 4). On the other hand, in patients with COPD, a lower MLD (MLDmax, MLD CR) results in a lower MPAD not only during exhalation but also during inhalation (*e.g.,* Fig. 5). Previous studies have shown a correlation between MLD and changes in total lung volume during inspiratory and expiratory CT [30], and one systematic review and meta-analysis showed an association between MLD and FEV_1_ predicted and FEV_1_/FVC in patients with COPD [31]. Thus, lower MLD was suggestive of expiratory impairment. The present study also showed a correlation between MLDmax and %RV in patients with COPD. Based on the above, it was considered reasonable to use the MLD of DVCT as a surrogate indicator of residual air volume in patients with COPD. In patients with COPD with more severe expiratory impairment, this may suggest that the effect of increased intrathoracic pressure contributes to reduced MPAD even during inhalation. Thus, MPAD in respiratory dynamics is affected not only by airflow limitation but also by expiratory impairment.

Based on this result that insufficient exhalation was associated with the reduction in expiratory MPAD, long-term standing exhalation impairment can lead to the remodeling of peripheral pulmonary arteries to some extent, due to lasting relatively higher intrathoracic pressure, although it should be reexamined for more cases in the future study. In addition, this unfavorable effect of decreased exhalation was observed even during inspiration for COPD patients. Therefore, we think that it is clinically important to pay attention to whether or not peripheral pulmonary arterial remodeling occurs or progresses for patients who were demonstrated to have exhalation impairment on pulmonary function test, especially for COPD patients, by checking it using a simple index such as trans tricuspid pressure gradient on echocardiography.

Previous reports have compared the systolic and diastolic diameters of the main pulmonary artery in cardiac motion, thereby assessing pulmonary arterial distensibility [3, 4, 6]. For example, Colin et al. have shown that the more advanced pulmonary hypertension due to COPD, the less distensibility it has [6]. However, these studies did not measure the blood vessels four-dimensionally in physiological breathing conditions, because the measurements were made with specific respiratory levels held, or even under respiration, without distinguishing between inhalation and exhalation, and may not reflect normal hemodynamics itself. In addition, each pulmonary artery diameter is a comparison of systolic and diastolic diameters in a single heartbeat and does not evaluate multiple consecutive heartbeats. In contrast, in the present study, the heartbeat-derived waves were isolated during respiratory dynamics, and the average of their amplitudes was taken as the amount of vascular CSA change during both systole and diastole. Therefore, it was possible to evaluate the average CSA change in all phases of physiological respiratory dynamics. Furthermore, comparison in the CSA change in each of the exhalation and inhalation phases is novel. Thus, while previous studies have evaluated only circulation, *i.e.,* right heart catheter data and rate of change in pulmonary artery diameter, this study evaluates the rate of change in diameter and respiratory function tests in respiratory kinetics and focuses more on the effect on the vessels in respiratory kinetics.

In this study, although there was a significant difference in age between patients with COPD and those without, there was no correlation between age and DCVT, Goddard Classification Score, or spirometry results, which may not have influenced the present study. On the other hand, there was also a significant difference in BMI between patients with COPD and those without COPD, but BMI was also positively correlated with total lung capacity, MLDmax, and MLDmin. This positive correlation between BMI and MLD is thought to reflect the decrease in body weight in association with hyperinflation generally observed in COPD patients. Generally, BMI should have a negative association with total lung capacity as well as vital capacity and FEV_1_ [32]. However, because no obese patients were included in this study, we speculate that muscle mass reflected in BMI may correlate positively with the total lung capacity.

There are several limitations to this study. First, DVCT in this study did not measure the heart rate during imaging, and the heart rate was calculated from the number of phases of DVCT and the number of waves caused by heartbeats on DVCT. However, the frequencies of heartbeat and respiration are quite different, and we believe that this information was sufficient to calculate the amplitude of the waves caused by heartbeat in the DFT. Second, the effect of data complementation on the quantification may be present because fourfold complementation data from a dedicated workstation (PhyZiodynamics; Ziosoft, Tokyo, Japan) was used to obtain the number of data samples needed for analysis by DFT. However, a similar study using this workstation has been reported, and we believe that the validity of the complementary data has been established [25]. This drawback may be overcome when DVCT with higher temporal resolution is developed with shortened rotation times in the future. Alternatively, we are considering future analyses for reconstructed image data with approximately half the temporal resolution of the present analysis using a half-reconstruction algorithm. Third, because most of the included patients underwent DVCT after contrast-enhanced CT examination as preoperative routine protocol and breathing practice, the time from injection of contrast media to the data acquisition of DVCT was different among the individual patients, and some of them underwent non-contrast CT. As a result, luminal attenuation in the MPA varied. As shown in the previous study, the association between CT attenuation value and measurement errors in the lumen and vessel wall [33] and the influence of the enhancement effect on the measurement of the luminal area cannot be denied. However, in this study, we tried to visually distinguish the right MPA from surrounding mediastinal adipose tissue and could trace it to the outer line of the vessel wall thanks to the distinct density difference between the adipose tissue and vessel wall, so the enhancement effect by the contrast agent is expected not to be so large.

In conclusion, we have shown that right MPAD during exhalation was reduced in patients with insufficient exhalation. Moreover, right MPAD decreased in association with a higher residual rate during inspiration as well as expiration in COPD patients. These results indicate that assessment of MPAD in different respiratory phases on DVCT can contribute to understanding the pathogenesis of pulmonary hypertension due to lung disease and/or hypoxia including COPD.

## Data Availability

The datasets used and/or analyzed during the current study are available from the corresponding author upon reasonable request.

## Abbreviations

COPD: Chronic obstructive pulmonary disease
CSA: Cross-sectional area
CT: Computed tomography
DFT: Discrete Fourier transformation
DVCT: Dynamic ventilation CT
FEV_1_: Forced expiratory volume in 1 s
FVC: Forced vital capacity
MLD: Mean lung density
MPA: Main pulmonary artery
MPAD: MPA distensibility
